# Multi-Omics Insights into Postnatal Skeletal Muscle Development in Duroc Pigs

**DOI:** 10.3390/ani15182715

**Published:** 2025-09-16

**Authors:** Kaiming Wang, Xin Li, Xibing Liu, Sui Liufu, Lanlin Xiao, Bohe Chen, Wenwu Chen, Jun Jiang, Yan Liu, Haiming Ma

**Affiliations:** 1 College of Animal Science and Technology, Hunan Agricultural University, Changsha 410128, China; 15116529648@stu.hunau.edu.cn (K.W.); 13243663120@163.com (X.L.); hunheshuiguocha@163.com (X.L.); liufusui0816@163.com (S.L.); finafantacyla@163.com (L.X.); chenhe0213914@163.com (B.C.); cww1242646778@163.com (W.C.); jiangjun1121@126.com (J.J.); 2 Tangrenshen Group Co., Ltd., Zhuzhou 412007, China; 3 Key Laboratory of Livestock and Poultry Resources (Pig) Evaluation and Utilization, Ministry of Agriculture and Rural Affairs, Changsha 410128, China; 4 Yuelushan Laboratory, Changsha 410128, China

**Keywords:** pig, skeletal muscle, transcriptome, metabolome, gene

## Abstract

Skeletal muscles, accounting for 40% of mammalian body mass, exhibit pronounced heterogeneity due to their distinct anatomical locations. Animal husbandry has focused excessively on *longissimus dorsi* (LDM) development while neglecting other muscles. In this study, we integrated Bulk RNA Sequencing (bulk RNA-seq) and Liquid Chromatography–Mass Spectrometry (LC-MS) analyses of *Soleus* (SOL), *Gastrocnemius* (GAS), and *Psoas major* muscles (PMM) across three key stages in Duroc pigs. We identified nine critical genes (*S100A1*, *MBOAT2*, *CA3*, *GYG2*, *ACTN3*, *ENO3*, *SLC3A2*, *SLC16A10*, and *GAPDH*) and eight metabolites potentially involved in regulating both skeletal muscle development and fiber-type transformation. The heterogeneity between SOL and GAS was low at birth but increased gradually during development. In contrast, PMM exhibited higher heterogeneity than SOL and GAS from birth. Notably, expression levels of *MYH7*, *MYH1*, and *MYH4* displayed stage-specific and muscle type-dependent variations. Moreover, we observed a developmental shift from the MAPK signaling pathway (1–21 d) to the regulation of the actin cytoskeleton (21–120 d). Pairwise comparisons between the SOL, GAS, and PMM revealed that the signaling pathways were enriched in muscle fiber-type switching. Collectively, through the integration of bulk RNA-seq and LC-MS data, this study provides novel molecular breeding strategies for the genetic improvement of meat-producing animals.

## Simple Summary

Skeletal muscles exhibit substantial heterogeneity in their developmental patterns and functions, depending on their location in the body. In this study, we identified six genes and eight metabolites that are potentially involved in regulating both skeletal muscle development and muscle fiber-type transformation. Notably, the divergence in SOL vs. GAS is minor at birth but increases gradually during development, whereas divergence in PMM vs. SOL and PMM vs. GAS is great from birth. Furthermore, we found that a developmental shift occurred from the MAPK signaling pathway (1–21 d) to the regulation of the actin cytoskeleton (21–120 d).

## 1. Introduction

In adult mammals, skeletal muscle accounts for approximately 40% of body weight. It is formed of highly heterogeneous and elastic tissues and plays a vital role in metabolism and movement [1]. Skeletal muscle development is mainly divided into two stages: embryonic and postnatal [2]. Skeletal muscle development requires myogenesis, a complex process regulated by numerous genes, including *LMOD2* [3,4]. Although transcriptomics (RNA-seq) has been extensively employed to identify genes involved in skeletal muscle development and regeneration, most studies are restricted to single anatomical sites. This is especially true in the field of animal husbandry, where most attention has been paid to the *longissimus dorsi* (LDM) and *soleus* (SOL), whereas other muscles have been overlooked [5].

Recent studies have focused on this topic. Terry et al. [6] used the bulk RNA-seq technique to measure more than 20 types of muscles in rats and mice. The results showed that more than 50% of the animal gene expression levels varied among the muscles. Notably, there is an average 13% difference in gene expression between any two muscle types. Jin et al. [7] revealed transcriptional differences among 47 skeletal muscles in pigs; however, this study did not uncover transcriptional variability at longitudinal developmental stages. Yang et al. [8] compared skeletal muscle transcriptomes across 27 developmental stages in Chinese and Western pigs, revealing the epigenetic mechanisms underlying the differences in muscle growth between the two breeds. However, this longitudinal study focused only on the LDM and lacked a cross-sectional assessment of distinct muscles. Given the limitations of these previous studies, we propose conducting a multi-muscle, multi-stage, and multi-omics study. Muscle aging is an inevitable process. The transcriptional profiles of 11 muscles in young and old mice indicated that the mitochondria-enriched SOL exhibited greater resistance to aging than other skeletal muscles [9]. Thus, an integrated longitudinal and cross-sectional analysis of distinct skeletal muscles is crucial for understanding the muscular system.

Skeletal muscle heterogeneity arises from the distinct composition of myosin heavy chain (MYHC) isoforms [10], which define phenotypic variations [11]. Postnatal development is a critical period for fiber-type transformation in pigs, as the dominance of oxidative fibers progressively declines with the expansion of glycolytic fibers between days 1 and 28 [12]. However, research on muscle fiber-type transformation has primarily focused on LDM, and the spatiotemporal dynamics of this transition across distinct muscles remain unclear. Comparative transcriptomics has revealed substantial expression divergence between oxidative (PMM) and glycolytic muscles (*Longissimus lumborum*, *Triceps brachii*, and *Semimembranosus*) in cattle, which reflects distinct metabolic programs [13]. Transcriptomic profiling has revealed that *Extensor digitorum longus* (EDL) and PMM are fast-twitch muscles, while SOL is a slow-twitch muscle [14]. The SOL displays significantly enriched slow-twitch fibers and intramuscular adiposity compared to adjacent hindlimb muscles. Notably, slow-twitch fiber density is positively correlated with intramuscular lipid accumulation, suggesting functional interdependence between these traits [15]. These results indicate that skeletal muscles at different anatomical locations have distinct structures, functions, and developmental patterns. However, cross-sectional and longitudinal multi-omics dynamics governing the development of distinct muscle types remain unclear.

In this study, in order to address these key issues, we conducted in-depth research on various types of skeletal muscles. We selected rapid-growth Duroc pigs as the model organism. We performed transcriptomic (Bulk RNA-seq) and metabolomic (LC-MS) profiling of porcine SOL (slow-twitch muscle), GAS (fast-twitch muscle), and PMM (fast-twitch muscle) at three key developmental stages (1, 21, and 120 d). Integrative analysis revealed the essential genes and metabolites governing skeletal muscle development and fiber-type transitions. This study provides novel directions and insights for the genetic improvement and breeding of meat-producing animals.

## 2. Materials and Methods

### 2.1. Experimental Animals and Cells

All experimental procedures in this study were approved by the Hunan Agricultural University Animal Care and Use Committee (permit number CACAHU-20250405). The experiment was conducted on a standardized farm where groups of six pigs were housed in pens. The ambient temperature and relative humidity were maintained at 25 ± 2 °C and 60–70%. Natural light was provided for 6 h daily. Pigs had free access to water and were fed a diet meeting NRC nutritional standards. Eighteen pigs were divided into three groups. The pigs in the same group were full siblings, while those in different groups were half-siblings. Three skeletal muscles (*Soleus*, *Gastrocnemius*, and *Psoas major*) were collected from 18 Duroc pigs at three developmental stages (1 d, 21 d, and 120 d; *n* = 6 per stage), snap-frozen in liquid nitrogen, and stored at −80 °C for subsequent transcriptomic and metabolomic analyses. C2C12 myoblasts (ATCC, New York, NY, USA) in proliferative (GM-cultured) and differentiated (DM-induced) states were harvested for subsequent RNA-seq analysis. The analysis of these cells is presented only as a supporting dataset and not as the primary focus of the study.

### 2.2. Total RNA Extraction and Quantitative Real-Time PCR

Total RNA was isolated from cells and tissues using TRIzol reagent (Invitrogen, Carlsbad, CA, USA). Total RNA was reverse-transcribed into cDNA using a kit (Invitrogen, Carlsbad, CA, USA). RT-qPCR was performed using the SYBR Green Kit (TransGen, Beijing, China), and the relative expression of genes was calculated using the 2^−ΔΔCt^ method. All primer sequences are listed in Table 1.

### 2.3. Western Blot Detection of Protein Expression

Total proteins were extracted from muscle tissues using RIPA lysis buffer (P0013B, Beyotime Biotech Inc., Shanghai, China) supplemented with protease and phosphatase inhibitors. Western blot analysis was performed with the following primary antibodies: MYH7 (1:1000, 22280-1-AP, Proteintech Group, Inc., Wuhan, China), MYH1 (1:10000, 67299-1-Ig, Proteintech Group, Inc., Wuhan, China), and Anti-GAPDH (1:10000, 10494-1-AP, Proteintech Group, Inc., Wuhan, China). Finally, protein bands were quantified using ImageJ 2 software.

### 2.4. Hematoxylin–Eosin (HE) Staining

Paraffin sections were deparaffinized, stained with hematoxylin and eosin, dehydrated, and mounted using an HE staining kit. Sections were then observed under a microscope, and images were captured for analysis. The muscle fiber cross-sectional area was quantified using ImageJ 2 (National institutes of Health), followed by a comparative analysis.

### 2.5. Immunofluorescence Assay

For immunofluorescence assays, fresh SOL, GAS, and PMM muscles were fixed in 4% paraformaldehyde and embedded in paraffin. Serial 20 μm sections were cut using a cryostat. Sections were incubated overnight at 4 °C with primary antibodies against fast-twitch (GB112130-100, Wuhan Servicebio Technology Co., Ltd., Wuhan, China) and slow-twitch (GB112131, Wuhan Servicebio Technology Co., Ltd., Wuhan, China) myosin heavy chains, followed by 1 h incubation with species-matched fluorescent secondary antibodies at room temperature. Images were acquired using a fluorescence microscope.

### 2.6. RNA-Seq and LC-MS Analyses

A total of 54 samples from three types of muscle tissues at three developmental stages (*n* = 6 per stage) were collected. Transcriptomic and metabolomic profiles were generated from these samples. RNA-seq and LC-MS analyses were performed by Shanghai Majorbio Bio-Pharm Technology Co., Ltd. (Shanghai, China). Specifically, to identify differentially expressed genes (DEGs) between the two groups, the abundance of each transcript was quantified based on the transcripts per million (TPM) method. RSEM was used to estimate transcript abundance. Subsequently, differential expression analysis was conducted using the DESeq2 and DEGseq packages. Transcripts exhibiting |log_2_FC| ≥ 1.5 and an FDR < 0.05 (for DESeq2) or FDR < 0.001 (for DEGseq) were defined as statistically significantly differentially expressed genes (DEGs). KEGG pathway analyses were performed using the Goatools and Python SciPy (1.14) packages. For LC-MS, variance analysis was performed on the preprocessed data matrix using the R package ropls (1.6.2). Principal component analysis (PCA) and orthogonal partial least squares-discriminant analysis (OPLS-DA) were conducted, and model stability was assessed using 7-cycle cross-validation. Significantly altered metabolites were selected based on variable importance in projection (VIP) scores from the OPLS-DA model (VIP > 1 and *p* < 0.05). Functional interpretation was performed using pathway enrichment analysis in the KEGG database.

### 2.7. Statistical Analysis of the Data

The qRT-PCR data are presented as means ± standard error. An unpaired Student’s *t*-test was performed between the treatment and control groups using GraphPad Prism 8, *, *p* < 0.05; **, *p* < 0.01.

## 3. Results

### 3.1. Phenotypic Assessment of SOL, GAS, and PMM in Duroc Pigs

**Figure 1:**
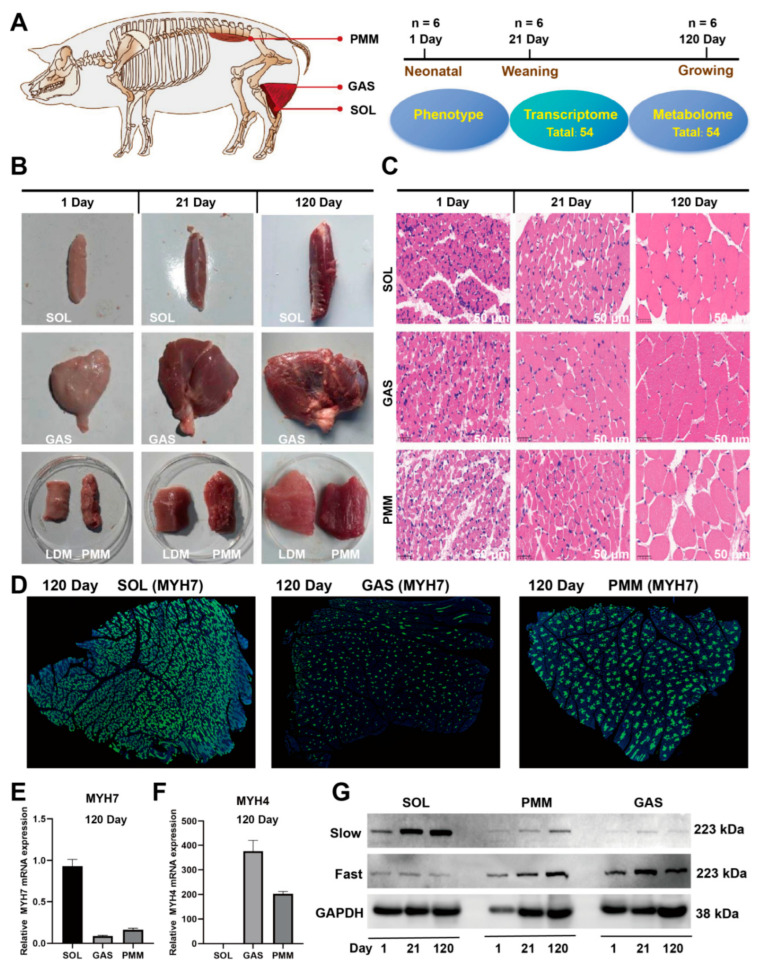
Phenotypic assessment of SOL, GAS, and PMM in Duroc pigs. (**A**) Experimental design diagram. (**B**) Phenotypic diagrams of SOL, GAS, LDM, and PMM are presented, with LDM as the control for PMM (*n* = 6). (**C**) HE staining of SOL, GAS, and PMM across three developmental stages (*n* = 6); Scale bar, 50 μm. (**D**) At 120 days of age, MYH7 antibody immunostaining was performed on SOL, GAS, and PMM (*n* = 3). (**E**,**F**) At 120 days of age, the mRNA expression levels of *MYH7* and *MYH4* in SOL, GAS, and PMM were detected using qRT-PCR (*n* = 3). (**G**) Protein expression levels of MYH7 and MYH1 during the development of the SOL, GAS, and PMM were analyzed by Western Blot (*n* = 3).

The picture presents anatomical, developmental, and experimental diagrams of SOL, GAS, and PMM in Duroc pigs (Figure 1A). The volume and weight of SOL, GAS, and PMM gradually increased as they grew (Figure 1B). Similarly, the muscle fiber cross-sectional areas of SOL, GAS, and PMM increased significantly (Figure 1C). At 120 days of age, immunofluorescence results demonstrated that slow-twitch muscle fibers in the SOL exhibited the highest expression compared to GAS and PMM (Figure 1D). At 120 days of age, we examined the mRNA expression of *MYH7* and *MYH4* in these three muscles. Results indicated that SOL primarily expressed *MYH7*, while GAS and PMM primarily expressed *MYH4* (Figure 1E,F). Furthermore, the MYH7 protein (slow-twitch) is mainly expressed in SOL, while the MYH1 protein (fast-twitch) is mainly expressed in PMM and GAS (Figure 1G). Collectively, these results confirm the accuracy of the sampling sites, and the inconsistent molecular expression in these three muscles during development can be further analyzed.

### 3.2. Identification of DEGs by Bulk RNA-Seq During Multiple Skeletal Muscle Development

**Figure 2:**
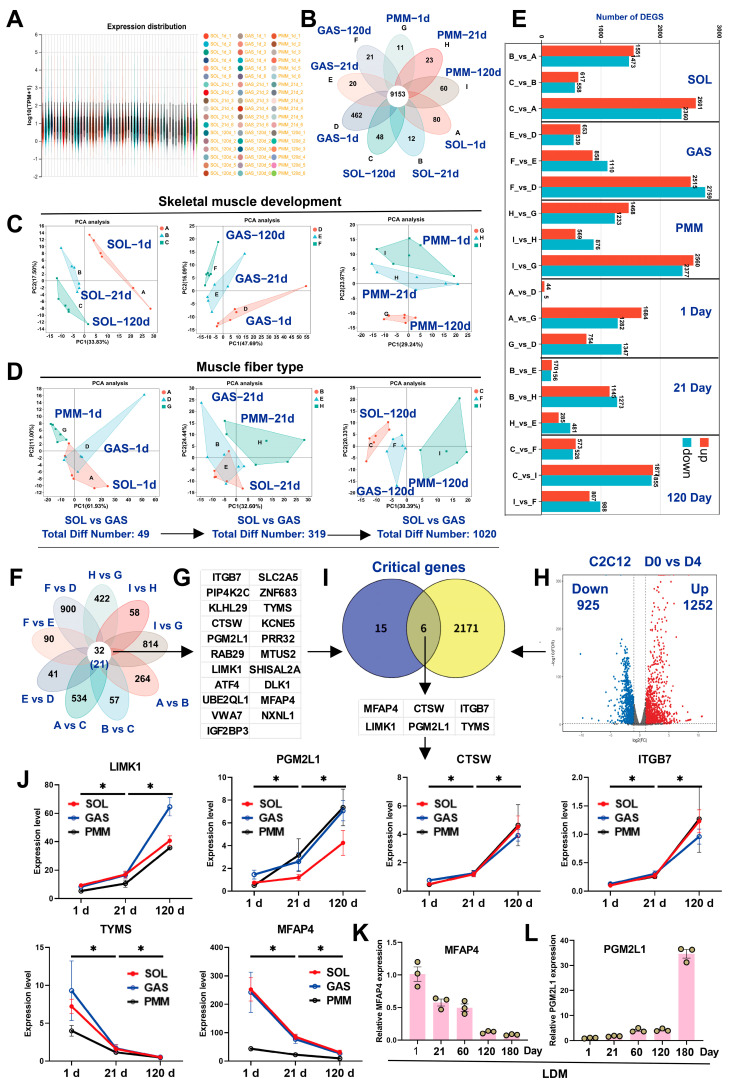
Identification of DEGs across multiple skeletal muscles using RNA-Seq. (**A**) Transcriptome-wide expression distribution in 54 muscle tissue samples. (**B**) Genes commonly expressed among the 54 muscle samples. (**C**,**D**) Principal Component Analysis (PCA) of skeletal muscle samples. (**E**) Number of DEGs between each pair of groups in the skeletal muscle samples. (**F**,**G**) Detected genes shared during longitudinal development in SOL, GAS, and PMM. (**H**) We performed transcriptome screening for DEGs between the proliferation and differentiation phases of C2C12 cells using data derived from our previous study [4]. (**I**) Six genes identified, which are shared between muscle development and C2C12. (**J**) Expression trends of six genes during three developmental stages in three muscle types. (**K**,**L**) Detection of the expression levels of *MFAP4* and *PGM2L1* genes during the development of LDM using qRT-PCR. * *p* < 0.05.

Transcriptomic analyses of SOL, GAS, and PMM across three developmental stages identified 9153 genes in 54 samples (Figure 2A,B). Principal Component Analysis (PCA) revealed differences among the three muscle types across the three developmental stages and exhibited strong intra-group clustering (Figure 2C). Notably, SOL and GAS muscles had only 49 differentially expressed genes (DEGs) on day 1 after birth, and the number of DEGs increased as the muscles developed. Neonatal transcriptomes of SOL and GAS were indistinguishable by PCA, indicating transcriptional similarity (Figure 2D). PMM exhibited significant differences in gene expression from both SOL and GAS after the postnatal stage (days 1–120) (Figure 2D,F). Venn diagram analysis identified 32 consistently shared genes across the SOL, GAS, and PMM at the three developmental stages, of which 21 were key genes (Figure 2F,G). The key genes mentioned in this study are listed in Table S1. Considering that myoblasts undergo proliferation and eventually differentiate into muscle fibers, we identified 2177 DEGs in the proliferation and differentiation stages of C2C12 (Figure 2H; see Table S5 for a complete list). Finally, six shared genes (*MFAP4*, *CTSW*, *ITGB7*, *LIMK1*, *PGM2L1*, and *TYMS*) were identified in muscle tissues and C2C12. These genes may regulate critical processes involved in skeletal myogenesis (Figure 2I). Among these six genes, four were progressively upregulated during development (days 1–120), while the remaining two were downregulated (Figure 2J). qRT-PCR validation of randomly selected *MFAP4* and *PGM2L1* across five developmental stages in the LDM muscle revealed expression patterns consistent with those of the SOL, GAS, and PMM muscles, confirming the reliability of transcriptome sequencing (Figure 2K,L). In conclusion, our experiments demonstrated transcriptional similarities between SOL and GAS muscles at birth, with progressive divergence during subsequent development. Additionally, six candidate genes were identified across longitudinal multi-muscle development.

### 3.3. Identification of DEGs by Bulk RNA-Seq in Fast-Twitch and Slow-Twitch Muscles

**Figure 3:**
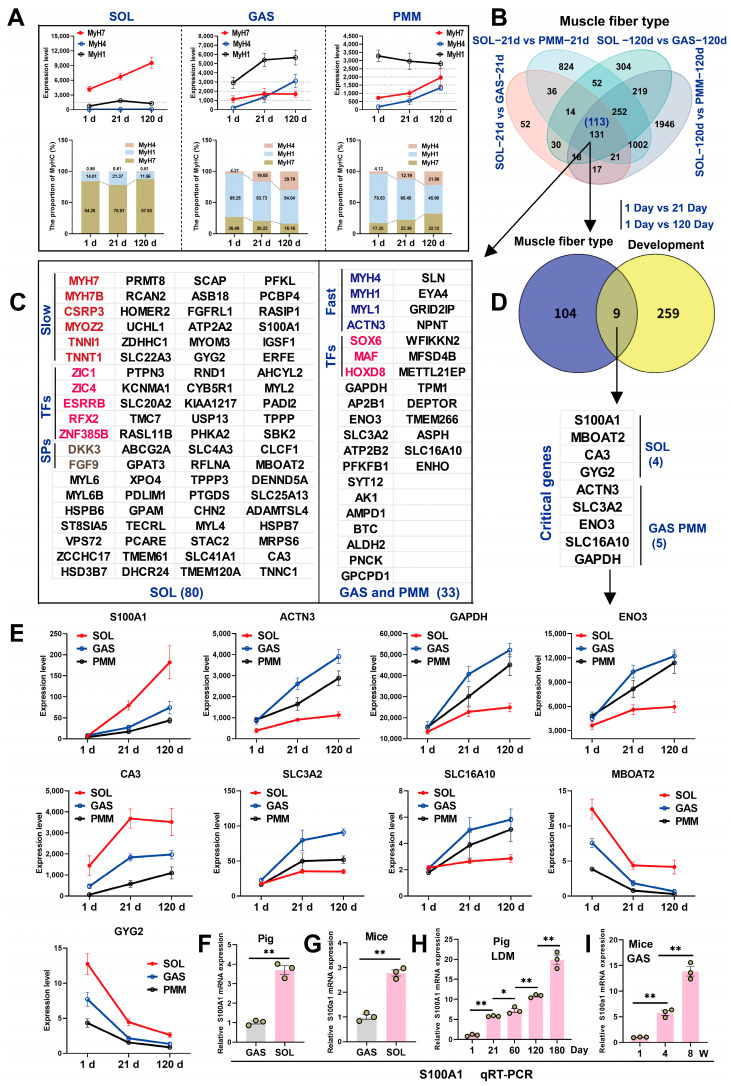
Identification of DEGs by bulk RNA-seq in fast-twitch and slow-twitch muscles. (**A**) Transcriptional expression levels of MYHC isoforms tracked across three developmental stages in SOL, GAS, and PMM. (**B**) Screening of DEGs in fast-twitch versus slow-twitch muscles using Venn diagrams. (**C**) Overall, 80 genes are highly expressed in the SOL, while 33 genes are highly expressed in both the GAS and PMM. (**D**) Genes identified to be shared between skeletal muscle fibers and multiple muscle development. (**E**) Expression trends of nine genes during three developmental stages in three muscle types. (**F**,**G**) *S100A1* expression levels were detected by qRT-PCR in the GAS and SOL of both pigs and mice. (**H**,**I**) *S100A1* expression levels were detected by qRT-PCR across five developmental stages in porcine LDM and three developmental stages in mouse GAS (1, 4, and 8 weeks). * *p* < 0.05; ** *p* < 0.01.

Transcriptome analysis revealed muscle-specific developmental dynamics of MYHC isoforms across different skeletal muscle types. In SOL, MYHC isoform proportions follow the order *MYH7* > *MYH1* > *MYH4*, with *MYH7* expression progressively increasing during development (days 1–120). In GAS, *MYH4* proportion progressively increases during development, whereas the proportions of *MYH1* and *MYH7* decrease. In PMM, MYHC isoform proportions follow the order *MYH1* > *MYH7* > *MYH4*. During development, the expression of *MYH7 and MYH4* progressively increased, whereas that of *MYH1* decreased (Figure 3A). These results demonstrate that SOL comprises slow-twitch muscle fibers (*MYH7*), whereas GAS and PMM predominantly comprise fast-twitch muscle fibers (*MYH1*). We identified 131 DEGs between fast-twitch and slow-twitch muscles, of which 113 were annotated (Figure 3B; see Tables S2 and S3 for a complete list). Of these 113 genes, 80 were highly expressed in SOL, while the other 33 were highly expressed in GAS and PMM (Figure 3B). Notably, transcription factors (TFs) enriched in slow-twitch muscle include *ZIC1*, *ZIC4*, *ESRRB*, *ZNF385*, and *RFX2*, whereas *SOX6*, *MAF*, and *HOXD8* are predominant in fast-twitch muscle (Figure 3C). In addition, the secreted proteins (SPs) *DKK3* and *FGF9* are enriched in slow-twitch muscles. We identified nine shared genes related to muscle fiber types and development (Figure 3D), namely *S100A1*, *MBOAT2*, *CA3*, *GYG2*, *ACTN3*, *ENO3*, *SLC3A2*, *SLC16A10*, and *GAPDH* (Figure 3E). qRT-PCR validation revealed significantly higher *S100A1* expression in SOL than GAS of both pigs and mice (Figure 3F,G). The expression level of *S100A1* gradually increased with skeletal muscle development in pigs and mice (Figure 3H,I). The qRT-PCR results were consistent with those of RNA-seq.

### 3.4. LC-MS Reveals Critical Metabolites for Skeletal Muscle Development and Myofiber Transformation

**Figure 4:**
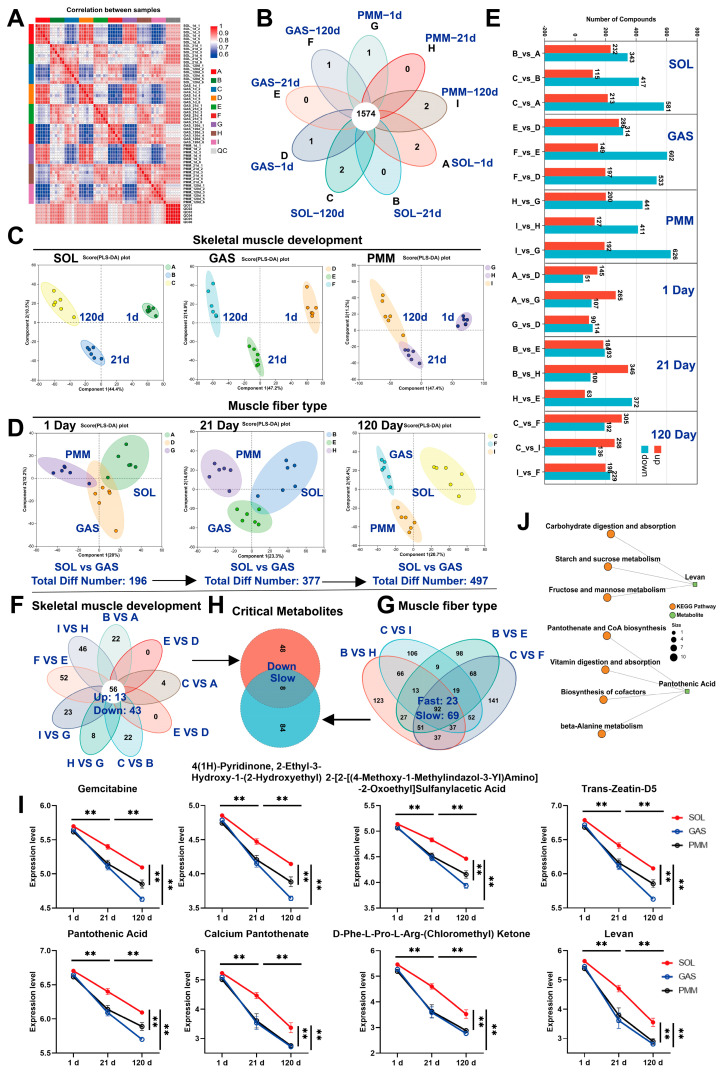
LC-MS analysis identified key metabolites governing skeletal muscle development and myofiber type transformation. (**A**) Inter-group correlation analysis of 54 muscle samples (nine groups, *n* = 6). (**B**) Venn diagram analysis of common metabolites across nine groups. (**C**,**D**) Partial Least Squares Discriminant Analysis (PLS-DA) of skeletal muscle samples. (**E**) Number of differential metabolites (DMs) between each pair of groups (*n* = 6) in the skeletal muscle samples. (**F**) DMs shared across longitudinal development in SOL, GAS, and PMM muscles identified using Venn diagrams. (**G**) Metabolites differentially abundant in fast-twitch and slow-twitch fibers identified. (**H**) Eight metabolites associated with muscle development and fiber-type transformation identified. (**I**) Expression trends of eight metabolites during three developmental stages in three muscle types. (**J**) KEGG pathways of metabolites pantothenic acid and levan. ** *p* < 0.01, *** *p* < 0.001.

We conducted LC-MS analysis on 54 samples, and the results showed that the reproducibility among the samples met expectations and could be used for subsequent analyses (Figure 4A). In these samples, 1574 common metabolites were identified (Figure 4B). Partial Least Squares Discriminant Analysis (PLS-DA) revealed differences among the three muscle types across three developmental stages, and strong intra-group clustering (Figure 4C). Notably, SOL and GAS muscles had only 196 differentially expressed metabolites (DMs) on day 1 after birth, and the number of DMs increased as the muscle developed (days 1–120). The PLS-DA model showed a gradual separation between SOL and GAS over time (Figure 4E). PMM exhibited significantly different metabolites compared to both SOL and GAS after the postnatal stage (Figure 4D). Venn diagram analysis identified 56 metabolites shared by SOL, GAS, and PMM across the three developmental stages. Among these, 13 metabolites were persistently upregulated and 43 were persistently downregulated (Figure 4F). We identified 92 DMs between fast-twitch and slow-twitch muscles. Among them, 69 DMs were enriched in slow-twitch muscle, and 23 DMs were enriched in fast-twitch muscle (Figure 4H). Ultimately, we identified eight crucial metabolites from the study of skeletal muscle development and muscle fiber type (Figure 4G).

Notably, these eight metabolites are enriched in slow-twitch muscle, with their abundance progressively declining during development (Figure 4I), and we have described the KEGG Pathway of Pantothenic Acid and Levan (Figure 4J). The key metabolites mentioned in this study are listed in Table S4. In conclusion, our experiments demonstrated metabolic similarities between SOL and GAS muscles at birth, with progressive divergence during subsequent development. Additionally, eight candidate DMs were identified. 

### 3.5. Analysis of KEGG Pathway Enrichment in Different Stages of Muscle Development

**Figure 5:**
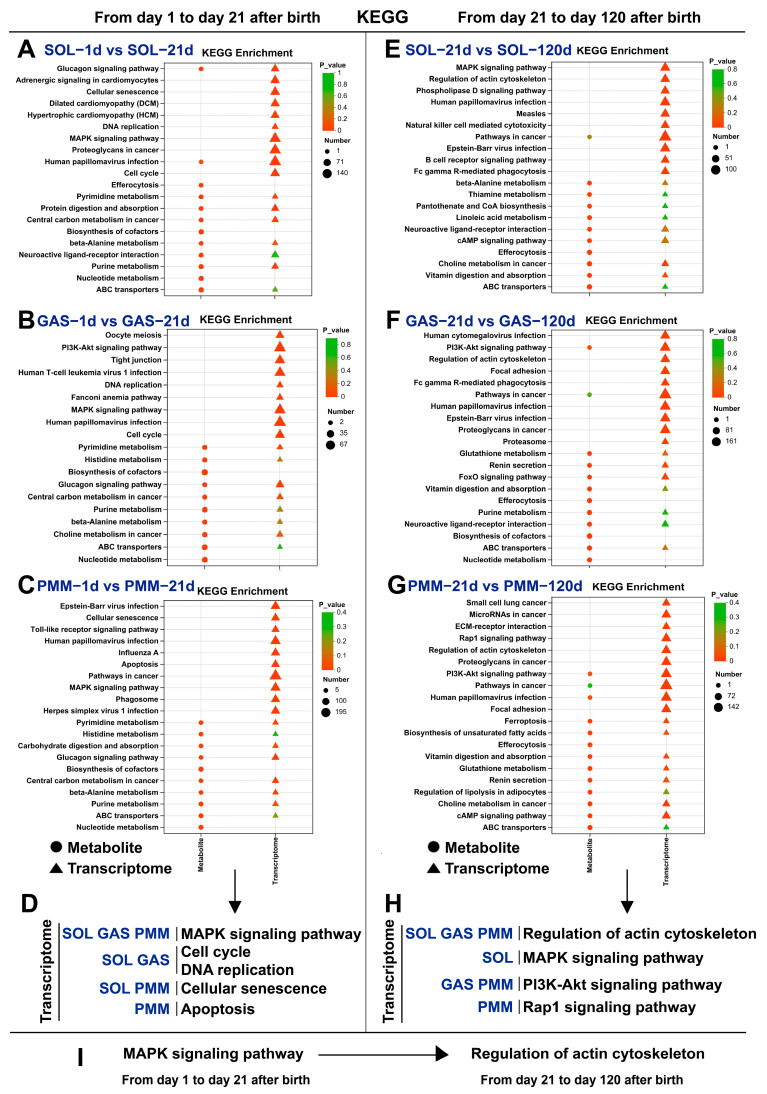
Integrated bulk RNA-seq and LC-MS data revealed KEGG pathways enriched in muscle development. (**A**–**C**) From postnatal days 1 to 21, the top ten enriched KEGG pathways in the SOL, GAS, and PMM were identified. (**D**) Important KEGG pathways are summarized. (**E**–**G**) From postnatal day 21 to day 120, the top ten enriched KEGG pathways in the SOL, GAS, and PMM muscles were identified. (**H**) Important KEGG pathways are summarized. (**I**) Transformation of KEGG pathways during the two developmental stages is summarized.

The Kyoto Encyclopedia of Genes and Genomes (KEGG) is a comprehensive pathway database enabling the functional annotation of gene and metabolite regulatory networks. The pig growth period was divided into two developmental stages: birth to weaning (days 1–21) and weaning to rapid growth period (days 21–120). Given the limited number of available enriched metabolites, we prioritized the analysis of gene-enriched KEGG pathways. Notably, at the first developmental stage (days 1–21), DEGs across all three muscle types showed co-enrichment in MAPK signaling, cell cycle, and apoptosis pathways (Figure 5A–D). During the second developmental stage (days 21–120), DEGs in all three types of skeletal muscles were enriched in the regulation of the actin cytoskeleton pathway, while fast-twitch muscles (GAS and PMM) exhibited enrichment in the PI3K-Akt signaling pathway (Figure 5E–H). In conclusion, our findings demonstrate a developmental shift from the MAPK signaling pathway to actin cytoskeleton regulation pathways during muscle development (Figure 5I).

### 3.6. Analysis of KEGG Pathway Enrichment in Different Muscles

**Figure 6:**
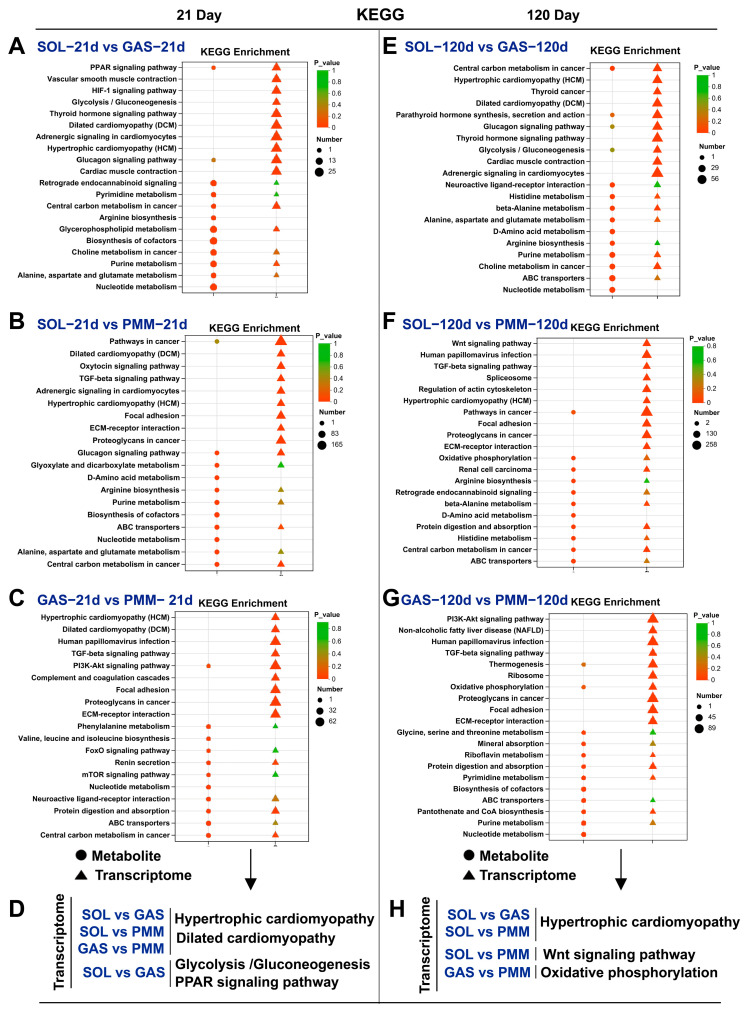
Integrated bulk RNA-seq and LC-MS data revealed KEGG pathways enriched in distinct muscle types. (**A**–**C**) KEGG signaling pathways enriched by DEGs and DMs from pairwise comparisons among the three muscle types on postnatal day 21. (**D**) The important KEGG pathways are summarized. (**E**–**G**) KEGG signaling pathways enriched by DEGs and DMs from pairwise comparisons among the three muscle types on postnatal day 120. (**H**) The important KEGG pathways are summarized.

Given the limited available enriched metabolites, we prioritized the analysis of gene-enriched KEGG pathways. On postnatal day 21, DEGs in slow-twitch (SOL) and fast-twitch (GAS and PMM) muscles were predominantly enriched in two pathways: hypertrophic cardiomyopathy (HCM) and dilated cardiomyopathy (DCM). Notably, DEGs in SOL versus GAS comparisons were significantly enriched in the glycolysis/gluconeogenesis and PPAR signaling pathways (Figure 6A–D). On postnatal day 120, DEGs in slow-twitch (SOL) and fast-twitch (GAS and PMM) were enriched in the HCM pathway (Figure 6E–H). Furthermore, DEGs between SOL and PMM were enriched in the Wnt signaling pathway (Figure 6H). We observed differences between the two fast-twitch muscles (GAS and PMM). On postnatal day 21, the DEGs were enriched in the HCM and DCM pathways. On postnatal day 21, the DEGs were enriched in the oxidative phosphorylation pathway (Figure 6D,H). In conclusion, our findings revealed differences among the three muscle types. Notably, although both PMM and GAS are fast-twitch muscles dominated by *MYH1*, distinct transcriptional profiles persist.

## 4. Discussion

Skeletal muscle development is divided into two stages: embryonic and postnatal. The embryonic stage determines the number of muscle fibers, whereas the postnatal stage relies on muscle hypertrophy [16]. Most studies have focused on temporal dynamics within single skeletal muscles or spatial comparisons across anatomical regions, but have not constructed spatiotemporal developmental maps of skeletal muscles. We hypothesized that skeletal muscle heterogeneity arises from postnatal developmental trajectory. The phenotypic results of the three skeletal muscles further confirmed this assumption. HE staining showed an increase in muscle cross-sectional area, and the pigs were in a healthy and rapid growth stage, making them ideal for our subsequent sequencing requirements. Given the close relationship between MYHC isoforms and meat quality, we first focused on their changes. The results revealed different expression patterns: the SOL predominantly expressed *MYH7*, while GAS and PMM primarily expressed *MYH1*. However, this finding does not fully align with that of a previous study in mice [14]. Furthermore, *MYH7* expression was higher in PMM than in GAS. Based on the slow-twitch fiber proportion, we believe that the meat quality ranks in the following order: SOL > PMM > GAS. Among the three muscle types, we identified key differentially expressed genes that could serve as targets for gene editing to increase the slow-twitch muscle proportion and thereby improve meat quality.

Based on MYHC isoform expression, we classified the skeletal muscles into slow-twitch SOL (*MYH7*-dominant) and fast-twitch GAS and PMM (*MYH1*-dominant), which is consistent with previous classification methods [7]. In contrast to other studies [17], this study profiled longitudinal MYHC isoform expression across three muscles. *MYH7* expression in SOL continuously increased, while that of *MYH4* was barely detectable. The GAS exhibits an expression pattern similar to that of the LDM [18], with a decreased proportion of oxidative muscle and an increased proportion of glycolytic muscle. PMM is one of the tenderest porcine muscles and has exceptional commercial value. In particular, we focus on PMM. During PMM development, both *MYH7* and *MYH4* showed increased expression and proportions, whereas *MYH1* expression decreased, with *MYH7* consistently exceeding *MYH4.* This MYHC isoform expression pattern during PMM development has not been previously reported. By integrating DEGs related to both skeletal muscle development and myoblast differentiation, we identified six genes (*MFAP4*, *CTSW*, *ITGB7*, *LIMK1*, *PGM2L1*, and *TYMS*) whose functional roles remain uncharacterized. We identified five transcription factors (TFs) enriched in slow-twitch muscle and three TFs (*SOX6*, *MAF*, and *HOXD8*) enriched in fast-twitch muscles. These TFs may be key factors distinguishing different muscle types. *SOX6* and *MAF* are closely associated with glycolytic muscle [19,20], while *HOXD8* shows differential expression in the GAS and SOL of mice [21], consistent with our results. Finally, nine DEGs were identified across muscle types and developmental stages. We focus on four genes related to slow-twitch muscle: *S100A1* and *CA3* are highly expressed in SOL, consistent with reports [22,23], whereas the values for *MBOAT2* and *GYG2* are unreported. The functions of these four genes require further investigation.

The metabolic capacity of skeletal muscles varies with developmental stage and anatomical location. In addition, metabolism varies among different pig breeds. Previous studies have identified the critical intramuscular fat deposition window by comparing the developmental stages of obese and lean pigs. Obese pigs exhibit higher energy expenditure and more active protein metabolism [24]. Using conventional LC-MS, we identified DMs at different developmental stages and in different muscle fiber types. Notably, the number of DMs in SOL and GAS gradually increased with development. This might be due to their different postnatal functions: SOL mainly maintains venous return, while GAS participates in explosive movements. Ultimately, we identified eight DMs involved in both skeletal muscle development and muscle fiber-type transformation. For example, pantothenic acid serves as a coenzyme A (CoA) precursor, which is implicated in energy metabolism and fatty acid oxidation [25], while levan polysaccharide exhibits potent anti-inflammatory and antioxidant activities [26]. Thus, these metabolites may serve as key regulatory factors influencing meat quality, but require further functional validation.

From postnatal days 1 to 21, the DEGs across all three muscles exhibited enrichment in the MAPK signaling pathway. The mitogen-activated protein kinase (MAPK) signaling pathway does not act independently [27]; instead, it forms regulatory networks with other pathways (e.g., p38 MAPK and PI3K-Akt pathways) to coordinate key physiological processes, including satellite cell proliferation and differentiation, muscle fiber hypertrophy, and fiber-type transformation [28,29]. Notably, over 60% of the genes enriched in the MAPK signaling pathway showed developmental upregulation (days 1–21); for instance, the *ATF4* gene is vital for skeletal muscle development [30,31,32]. In addition, cell cycle-enriched genes in SOL (86.5%) and GAS (75.6%) showed predominant developmental downregulation, whereas apoptotic-enriched genes in PMM (59.2%) were upregulated. These findings demonstrate that satellite cell cycle exit initiates myofiber differentiation, which is a prerequisite for skeletal muscle development [33]. From postnatal days 21 to 120, the DEGs in SOL, GAS, and PMM exhibited significant enrichment in the regulation of the actin cytoskeleton pathway. Studies have shown that this pathway plays a crucial role in muscle cell fusion, sarcomere assembly, and differentiation of muscle fiber types [34]. Fast-twitch muscles (GAS and PMM) showed PI3K-Akt pathway enrichment (days 1–120), whereas slow-twitch SOL exhibited MAPK pathway enrichment. This pattern may be linked to differential MYHC expression trends, as the MAPK pathway mainly drives slow-twitch formation [35], while PI3K-Akt mediates fast-twitch hypertrophy. In summary, during longitudinal development, SOL, GAS, and PMM exit the cell cycle and initiate differentiation first, but subsequently diverge from each other. Despite both being fast-twitch muscles, PMM and GAS showed significant divergence in KEGG, which is worth discussing in detail. On postnatal day 21, DEGs were enriched in DCM and HCM signaling pathways, and by day 120, enrichment shifted to oxidative phosphorylation. The PI3K-Akt pathway was enriched at both time points. This may be because the PMM has more slow-twitch muscle fibers than the GAS. Early studies have defined PMM as an oxidative muscle [36]. This study demonstrated high similarity between SOL and GAS in newborns, which can be attributed to their anatomical proximity. However, whether this pattern applies to other hindlimb muscles still needs to be verified.

The limitations of this study are that it only focused on three muscles and three developmental stages, and only employed conventional bulk RNA-seq and LC-MS techniques. Future work should integrate multiple omics (e.g., single-cell RNA sequencing, spatial transcriptomics, and ATAC sequencing) to explore the molecular mechanisms of the four species, 20 muscle types, and 15 developmental stages.

## 5. Conclusions

This study identified six genes and eight metabolites that are key regulators of skeletal muscle development and fiber-type transformation. SOL and GAS heterogeneity were low at birth but increased during development. *MYH7*, *MYH1*, and *MYH4* expression varied according to the stage and muscle type. A developmental shift occurred from the MAPK signaling pathway (1–21 d) to actin cytoskeleton regulation (21–120 d). These results provide a theoretical basis for livestock improvement.

## Figures and Tables

**Table 1:** The primer sequences used in this study.

Species	Genes	F: Sequence (5′ to 3′)	R: Sequence (5′ to 3′)
Pig	*MYH1*	GAAGTTGCATCCCTAAAGGCAG	CGATGACTTGGCGTCAAAAGG
Pig	*MYH2*	GGAGGCTGAGGAACAATCCA	GCATCGGGACAGCCTTACTC
Pig	*MYH4*	AGTTCCGTAAGATCCAGCACG	CCTGTCACCTCTCAACAGAAAGA
Pig	*MYH7*	AGTCCCAGGTCAACAAGCTG	TTCCACCTAAAGGGCTGTTG
Pig	*MFAP4*	CGGCGTGTACCTCATCTACC	GCCGTTGAACCTCTTCTGGA
Pig	*PGM2L1*	CGTCTTTTCACGGAGTCGGA	TGAAGTTCTGCCACTGCCAA
Pig	*S100A1*	GAACTGGAGACAGCGATGGA	TTGTTACAGGCCACTGTGAGG
Mouse	*S100A1*	AGAGTGCCATGGAGACCCTC	GCTCAACTGGTCTCCCAGAA
Pig, Mouse	*GAPDH*	AGGGCATCCTGGGCTACACT	TCCACCACCCTGTTGCTGTAG

## Data Availability

The raw Bulk RNA-seq data are available in the NCBI database (Accession: PRJNA1321496), and the raw LC-MS data are available in the MetaboLights database (Accession: MTBLS12963). All raw data are also available from the corresponding author upon request.

## Supplementary Materials

The following are available online at https://www.mdpi.com/article/10.3390/ani15182715/s1, Table S1: Differentially expressed genes of SOL, GAS, and PMM during the three developmental stages. Table S2: Differences in genes between different muscle fiber types: SOL compared with GAS and PMM. Table S3: Differential genes between GAS and PMM. Table S4: Key metabolites mentioned in this study. Table S5: Differentially expressed genes between C2C12 proliferation and differentiation [4]. Abbreviations of column headers in tables: FC, fold change; Log2FC, log2 fold change; Padjust, adjusted *p*-value (Benjamini-Hochberg FDR); *, *p* < 0.05; **, *p* < 0.01. Significantly differentially expressed genes were defined as those with |Log2FC| ≥ 1.5 and *p* < 0.05, and were classified as upregulated (Log2FC > 0) or downregulated (Log2FC < 0).
